# Treatment-Seeking for Tuberculosis-Suggestive Symptoms: A Reflection on the Role of Human Agency in the Context of Universal Health Coverage in Malawi

**DOI:** 10.1371/journal.pone.0154103

**Published:** 2016-04-21

**Authors:** Moses Kumwenda, Nicola Desmond, Graham Hart, Augustine Choko, Geoffrey A. Chipungu, Deborah Nyirenda, Tim Shand, Elizabeth L. Corbett, Jeremiah Chikovore

**Affiliations:** 1 Malawi Liverpool Wellcome Research Programme, Blantyre, Malawi; 2 Helse Nord TB Initiative, College of Medicine, Blantyre, Malawi; 3 Liverpool School of Tropical Medicine, Liverpool, United Kingdom; 4 School of Life & Medical Sciences, University College London, London, United Kingdom; 5 London School of Hygiene and Tropical Medicine, London, United Kingdom; 6 School of Public Health and Family Medicine, College of Medicine, Blantyre, Malawi; 7 Institute for Global Health, University College London, London, United Kingdom; 8 School of Public Health and Family Medicine, University of Cape Town, Cape Town, South Africa; 9 HIV/ AIDS, Sexually Transmitted Infections & TB, Human Sciences Research Council, Durban, South Africa; Universidad Nacional de la Plata, ARGENTINA

## Abstract

Tuberculosis (TB) is highly infectious and one of the leading killers globally. Several studies from sub-Saharan Africa highlight health systems challenges that affect ability to cope with existing disease burden, including TB, although most of these employ survey-type approaches. Consequently, few address community or patient perspectives and experiences. At the same time, understanding of the mechanisms by which the health systems challenges translate into seeking or avoidance of formal health care remains limited. This paper applies the notion of human agency to examine the ways people who have symptoms suggestive of TB respond to and deal with the symptoms vis-à-vis major challenges inherent within health delivery systems. Empirical data were drawn from a qualitative study exploring the ways in which notions of masculinity affect engagement with care, including men’s well-documented tendency to delay in seeking care for TB symptoms. The study was carried out in three high-density locales of urban Blantyre, Malawi. Data were collected in March 2011 –March 2012 using focus group discussions, of which eight (mixed sex = two; female only = three; male only = three) were with 74 ordinary community members, and two (both mixed sex) were with 20 health workers; and in-depth interviews with 20 TB patients (female = 14) and 20 un-investigated chronic coughers (female = eight). The research process employed a modified version of grounded theory. Data were coded using a coding scheme that was initially generated from the study aims and subsequently progressively amended to incorporate concepts emerging during the analysis. Coded data were retrieved, re-read, and broken down and reconnected iteratively to generate themes. A myriad of problems were described for health systems at the primary health care level, centring largely on shortages of resources (human, equipment, and drugs) and unprofessional conduct by health care providers. Participants consistently pointed out how the problems could drive patients from promptly reporting symptoms at primary healthcare centres. The accounts suggest that in responding to illness symptoms including those suggestive of TB, patients navigate their options taking into cognisance past and current experiences with formal health systems. Understanding and factoring in the mediating role of such ‘agency’ is critical when implementing efforts to promote timely response to TB-suggestive symptoms.

## Introduction

Tuberculosis (TB) remains a major public health problem and is the leading cause of morbidity and mortality worldwide, despite progress that includes reversing upward trends in TB incidence [1, 2]. In 2013, the African region, home to 12% of the global population, accounted for 29% of cases, coming second only to South Asia with 56%. In high HIV prevalence settings such as the southern African region, the vulnerability of people living with HIV (PLWH) to rapid progression to TB following infection makes early diagnosis and treatment especially important. According to the 2014 global TB report, 13% of people who developed TB in 2014 were infected with HIV [1]. HIV has dramatically reversed the downward trend of TB in sub-Saharan Africa in the recent past [3–5]. The African region accounts for 80% of HIV-positive TB cases and TB fatalities among HIV infected people [1]. In Malawi specifically, case notification has increased fivefold between 1984 and 2006 [6], trailing the accelerated growth in HIV burden on the continent.

There is currently a steady but slow decline in TB incidence, prevalence and mortality within the African region[1]. Among factors that hamper TB control efforts are weak health systems, whose effects include poor infection control and prevention practices, inadequate supply chain management leading to stock-out of diagnostic and treatment essentials, and low quality of care as decided by patients [7–9]. In addition, End TB, the global TB control strategy currently in use in many countries including Malawi, relies primarily on individuals presenting at a health care facility rather than undertaking active case finding in the community where transmission occurs.

In turn, at the level of the community, delayed treatment-seeking for symptoms suggestive of TB arises from inappropriate health care-seeking habits, which are shaped by socio-economic factors such as poverty, the financial burden of diagnosis and treatment on patients, and associations made between TB and HIV [7–9]. Moreover, there is a gendered pattern in TB epidemiology; men in Africa generally have a higher burden of both undiagnosed and diagnosed TB [10–14]. This may reflect the interplay between higher true incidence of disease in men, and their lesser engagement with primary health care (PHC) and longer delay in seeking care for most illnesses including for TB [15–19]. In Africa, poverty also seems a more critical barrier to treatment seeking than gender [7, 20–28], compared to many parts of Asia where, to a large extent, gender disparities explain women’s longer diagnostic delay including at facilities [29–31].

Stemming the spread of TB requires action at different levels, including facilitating early case detection through interventions that take into account determinants of health services utilisation at community and health care provision levels. It is critical to understand health care systems problems, as well as how TB disease symptoms are recognised and acted on [32–34]. This paper draws on perspectives generated from ordinary community members, TB patients, undiagnosed chronic coughers, and health care workers to understand the interaction of PHC facilities with patients and the community. The perspectives emerged within a larger qualitative study that sought to understand why men remain undiagnosed with TB in the community generally, but more specifically when having symptoms suggestive of TB [33]. During the study, it emerged health systems challenges were a major concern that likely affected men and women’s ability and willingness to engage with formal health care services. This paper highlights the health systems challenges described, and the implications for health care seeking by men and women, and conceptually links these by applying the notion of *human agency*.

### Human agency

According to Emirbayer and Mische [35], human agency refers to “the temporally constructed engagement by actors of different structural environments—the temporal- relational contexts of action—which, through the interplay of habit, imagination, and judgment, both reproduces and transforms those structures in interactive response to the problems posed by changing historical situations” [35]. There are three constitutive elements to it, i.e. past (iterational), future (projective), and present (practical-evaluative). The iterational element entails the selective reactivation of ‘past patterns of thought and action, as routinely incorporated in practical activity, thereby giving stability and order to social universes and helping to sustain identities, interactions, and institutions over time’ [35]. The projective element involves ‘imaginative generation by actors of possible future trajectories of action, in which received structures of thought and action may be creatively reconfigured in relation to actors’ hopes, fears, and desires for the future’ [35]. Lastly, the practical-evaluative element involves the ability of actors ‘to make practical and normative judgments among alternative possible trajectories of action, in response to the emerging demands, dilemmas, and ambiguities of presently evolving situations’ [35]. Any concrete empirical case or action involves all three elements in varying degrees of magnitude, but one becomes dominant in defining and so defines too how a social actor relates to the other two at any given point.

This paper holds that the decision to seek formal health care for symptoms suggestive of TB is a process that involves patients, as social actors or agents, weighing the costs and benefits of available options by negotiating and navigating past experiences, present realities and possible future trajectories.

## Methods

Table 1 summarises the data gathering techniques and the study participants and their characteristics [33]. Data were collected over 12 months from March 2011, in three high-density and low-income suburbs of urban Blantyre in Malawi. Given limited understanding around the study topic, purposive sampling [36] was employed. The aim was to include participants best placed to provide information, and to vary sources of data to allow exploring different dimensions and generating a comprehensive picture of the study issue, as well as enhancing truthfulness and completeness [37] through participant and data techniques triangulation [38, 39].

**Table 1:** Data collection techniques, study participants and sample size.

Technique	Participant category	N (total participants)	Gender
FGD	HCW	2 (20)	Mixed sex (3men and 7 women per group)
	Community members	8 (74)	3—men only; 3 women only; 2- mixed (11 women, 9 men)
IDI	New TB patients	20	6 men; 14 women
	Chronic coughers	20	12 men; 8 women

In-depth interviews (IDIs) (n = 20; female = 14) were conducted with patients who had been diagnosed with TB in the month preceding their being contacted, to understand patients’ personal experiences of symptoms and care seeking. Patients were identified through TB registers and TB cards from two primary health centres, and traced using an approach similar to the one routinely used to follow-up TB patients; specifically, through their physical addresses or cell-phone numbers. Additional IDIs (n = 20; female = 8) were carried out with un-investigated chronic coughers to explore meanings given to symptoms suggestive of TB, and the associated care-seeking practices. Participants were identified through cluster enumeration from a TB/HIV epidemiology cluster randomised trial (CRT) also conducted in the study catchment area. The CRT investigated, among other things, the presence of cough with duration of three weeks or more within enumerated households [40–42].

Focus group discussions (FGD) were held with 74 members of the community (n = 8; Male only = 3; Female only = 3, Mixed sex = 2) to investigate general beliefs regarding health care seeking, TB and chronic cough, and normative views of gender roles. Potential participants were approached in their respective homes, informed about the study and invited to take part in the group discussions. Two FGDs with 20 health care providers were conducted to gain an understanding of the operations of the health delivery system and its interaction with patients and the community. Participants were identified at health service centres and comprised different cadres including nurse midwife technicians, nurses, health surveillance assistants, TB officers and clinical officers.

In short, the research process, which is described in greater detail in a related prior paper [43] was aligned to grounded theory as propounded by Charmaz [44]. Data were collected by two graduate level social scientists led by MK both of whom are fluent in Chichewa, with oversight from JC. None of the participants who were contacted and were eligible directly declined to participate; a sizeable number were excluded for not meeting inclusion criteria, while five TB patients could not be traced based on credentials that they had supplied to facilities. Fifteen ‘chronic coughers’ were excluded because they did not have a chronic cough at the time of our visit. All 96 potential participants invited to community FGDs accepted; however, 22 did not show up, as did two for the health care providers’ FGDs.

Data were recorded, and transcribed and translated verbatim by trained personnel, and then checked for accuracy by MK and intelligibility by JC. The transcripts were analysed using NVivo 9 data analysis software, with text being coded, retrieved and categorised and reconnected iteratively to generate and consolidate thematic categories. The coding scheme was developed both from the study aims and from themes that were emerging during the emergent analysis [37]. Coding was iteratively repeated until themes were saturated and refuting data had been taken into account. Health workers’ perspectives were examined to provide a validating perspective to the community and patient accounts. Parallel coding and analysis were done by MK and JC. Agency was then applied as an analytic lens to further make sense of the emerging themes.

The study was approved by College of Medicine Research Ethics Committee in Malawi and Human Sciences Research Council Research Ethics Committee in South Africa. Informed consent was obtained from all study participants, and the research team members observed and maintained anonymity and confidentiality of the study participants during the entire research process.

## Findings

Themes emerging from the analysis pointed to barriers to treatment that fell into two distinct yet interconnected domains, namely community as well as PHC system levels. Participants described myriad health systems challenges, among them equipment and drug shortages, and poor patient-health provider interaction, that led to general distrust of and lack of faith in the health system. Another prominent theme revolved around perceived unprofessional conduct by health care providers, including being generally disrespectful of patients and failing to uphold confidentiality of sensitive information. Views of community members and patients were confirmed by health care workers. The themes and perspectives are presented accompanied with verbatim quotes carrying limited participant identifying information.

### Lack of faith in primary health care system arising from poor communication and equipment and drugs shortage

Community members expressed frustration at what they saw as recurrent drug unavailability at primary health facilities. They repeatedly pointed to an uncertainty among patients that they would ever obtain drugs upon visiting a PHC centre; instead, patients would almost always be sent out to purchase prescribed drugs. Pointing to poor communication with regards to how healthcare workers may implement syndromic management, patients expressed strong views over the way drugs were prescribed. They felt that drugs were prescribed indiscriminately, with similar drugs being consistently prescribed across different patients or multiple visits by individual patients, and painkillers being given to ‘cure’ whatever problem patients presented with, all without carrying out medical investigations:

“You go and explain ‘I am having fever or I am vomiting’, they’ll not examine you. They just give you ***LA*** (artemether lumefentrine) without knowing what you could be suffering from. This discourages people from going there (to a PHC centre)” [Women’s FGD]

Perennial unavailability of drugs at PHC facilities even when the facilities had just had deliveries left patients wondering whether health personnel were misappropriating drugs either for resale or for use by themselves and their relatives. The feelings of bitterness that these thoughts and the uncertainty of drug availability evoked were exacerbated by considerations that when the drugs were thus not available, this was not communicated timeously to patients.

“…instead of telling you in good time that ‘there is no medicine’, they keep you until 12 midday. When you eventually go to the (facility) pharmacy, you are told there is no medicine, by which time you have already wasted valuable time that you could have used to go to another facility. In the end, you go home without treatment and continue suffering.” [Women’s FGD]

Reiterating the sentiment that necessary examinations and tests were often not done, participants described having to visit clinics on multiple occasions or having to physically deteriorate before any tests were initiated or they were accurately diagnosed. Partly to try and speed up the process, patients allegedly tried to instruct and advise health providers about which tests were appropriate for their own symptoms and health status, although this behaviour could offend the health care providers. On their side, health care providers acknowledged the delay patients experienced in obtaining a diagnosis even when they may have done their part by presenting early.

“Sometimes you come to the clinic promptly. If you have been coughing for three weeks and have a sputum test, you will be told that they did not find germs, but when you get home you are still not okay. In the long run, those germs could be multiplying, and that person will be seriously ill when TB is finally diagnosed.” [Woman, Community Nurse, Health care Worker FGD]

Further examples of poor interaction between health care providers and patients related to HIV testing which patients worried about, thinking it would be carried out without explanation or consent. HIV stigma is still widespread and leads to apprehension about provider initiated testing once TB has been diagnosed. Patients felt unable to question let alone object to health care provider recommendations to test for HIV, resulting in fear about contacting health services in the first place. Additionally, participants spoke of patients being rushed through impersonal consultations by health care providers, who showed little interest in obtaining a full account of patients’ presenting problems. Partly emphasising the concern expressed earlier in a women’s FGD, the following sentiments emerged in a FGD with men.

“When you are explaining, they finish writing in your *book* (health record) before you finish speaking. Cough, they write ***LA***, stomach-ache, ***LA***… backache, still ***LA*** for you. Any disease it is ***LA***… So when you fall sick, you ask yourself: ‘Should I go and receive that same ***LA***? Maybe I should not bother… even if it means dying at home.” [Men’s FGD]

A TB patient related her personal experiences in ways that illustrated how failure by health care workers to engage with patients also led to delay in getting a diagnosis. Despite making multiple visits to the PHC facility, she reported having been given only anti-biotics and painkillers, and health care providers investigating her for TB only when she showed serious signs of deterioration.

“…several times I went to a primary care centre to explain and was just given medicine and sent home. Sometimes they would give me ***amoxicillin***, sometimes ***bactrim***, sometimes some tiny pills… I wasn’t improving and had to go back to tell them that the medicine didn’t help, so they gave me different drugs. It wasn’t until I started coughing up blood that they found TB.” [Woman, TB patient, IDI]

The behaviour and attitude of health care providers, and how they seemed to lack time and patience, appeared to convince patients that their interests and individual health were not a concern to the health system. One chronic cougher related how, in fact, owing to bad experiences, she no longer had any interest in using the formal health care system:

“The problem I see in health centres is that when you are explaining that you have been coughing for a long period, they tell you to take medicine just in case it might work even when you tell them that you have been coughing for more than 3 weeks. Instead of helping you and collecting sputum to examine, you find that they start by giving you ***panado*** (painkiller). ‘Yea, go and take ***panado*** first, if there is no change, come back.’ When you come the second time, they will give you ***bactrim***. If there is no change, they just say ‘come again’. But during that time the person’s health is continuing to worsen.” [Woman, un-investigated chronic cougher: IDI]

### Rudeness and lack of confidentiality by health workers

Accounts chronicled what was deemed to be unprofessional and rude conduct by health care workers, with participants repeatedly recounting how they had been either simply ignored, or even taunted and humiliated. Having handheld health records thrown back by healthcare providers was cited by many participants. On some occasions, such events could occur within the presence of family members and other people known personally to the patient, irrespective of the respect the patient commanded or their status back in their own homes and communities.

“When you tell them ‘my body is aching’, they (healthcare providers) say: ‘What about your body ache, so you think that this is a place to stop that body ache of yours’? … Stomach pains, they say: ‘You are just constipated…’ then they throw your health passport at you (health record document).” [Women’s FGD]

The known association between TB and HIV and consequent stigma meant that people were concerned about the confidentiality of their diagnosis. However, according to our participants, there was a risk that health care providers would discuss or refer to sensitive health details, for example about TB, HIV or sexually transmitted infections, speaking loudly in front of other patients or in poorly sound-proofed consultation rooms. Health care providers were even said to gossip about patients’ medical conditions including at socialisation venues.

“They (health care providers) come and humiliate people in this very same village, saying things like: ‘Mr. so and so, he is “topping up” [i.e. prolonging life through ART, seen as similar to adding credits to a cell-phone account].” [Man, Mixed group FGD]

As PHC facilities tended to be congested, patients usually arrived early in order to be attended to earlier in the day. However, health care providers could appear overly relaxed and in no rush to get through the queue, instead chatting about issues unrelated to work before starting to serve patients. Community members described the hurtful, distressing and painful experience of having to wait long hours for consultations, with no formal queuing system, and while already in pain and also becoming increasingly weak from hunger.

“We feel reluctant to go (to the PHC facility) because even if we arrive at 5 o’clock in the morning, they open at 10 o’clock or maybe 11 o’clock. After waiting for a long time in the queue, they (health care providers) will just be helping their relatives … a person like me will be just watching others go without being at all noticed. Then I say, ‘It is better to just go and buy medicine at the market than be treated like that’.” [Women’s FGD]

Health care providers acknowledge the description of community members as accurate, and they were aware of many of the patients’ concerns. However, they pointed to the huge frustrations and constraints felt when faced with insufficient human resources, drugs, laboratory supplies and equipment, amid a growing demand for public health services. Irregular processing of their salaries also influenced their morale and attitude.

“…clinical instruments are scarce. A person who is coughing will go to the TB office and say: ‘I would like to be tested’, only to be told ‘slides finished last week, we are no longer testing’. If those people have not been assisted, will they come back for a test next week? They would obviously go to some other place, or maybe just stay put if they do not have money.” [Woman, Health Surveillance Assistant, Health Provider FGD]

“When the working conditions are not good, it causes providers to start acting as if they don’t like their job. Whenever they see a patient, they behave as if they [the patient] are causing their work conditions to be hard. They are, therefore, often harsh… because working conditions, including salaries, are not good. Worries such as about receiving salaries late, which often happens in the government sector, are transferred onto the patient. When you have not been paid, but still have to do a lot of work, you lose interest. ‘These patients are delaying me from making money elsewhere.’ These are the concerns that can make you act harshly.” [Man, Nurse Midwife Technician, Health Provider FGD]

## Discussion

Malawi has made commendable progress in the health sector by including PHC facilities within the hierarchy of health delivery in order to ensure free and accessible health care to the majority poor. Our findings point nevertheless to salient health system problems, as is the case in most resource-constrained settings [45, 46]. On the one hand is a frustrated health care workforce overburdened by workload, constrained by unavailability of essential resources, and disillusioned with their remuneration patterns, in ways well reported in the literature as interfering with optimum discharge of duties [45, 47]. Unsurprisingly, their frustrations were often vented on patients. While PHC staff admitted to unprofessional conduct and knew that it was improper, they did not consider some of their actions as unreasonable given the circumstances.

Then on the other hand is the materially deprived patient, and whose expectations about services offered at PHC centres are still barely met. The patient’s reaction includes taking conscious, though not entirely voluntary, decisions to procrastinate or completely defer seeking health care, or to purchase drugs from unlicensed vendors [32]. In instances where patients do engage with formal health care, they lack the confidence that they are receiving dedicated or even correct service. From a human agency theoretical perspective, therefore, community narratives of poor patient interaction with PHC services are an important indicator of how patients order and schematize previous and current experiences to generate an impression of expected future experiences.

In patients’ eyes, problems encountered are a common feature of PHC services. Communities and patients take cues, even on the basis of isolated negative incidents, to re-affirm their general perceptions of health services, with enduring effects on subsequent treatment seeking behaviour [48, 49]. The capacity to remember, select and apply schemas of action developed through past interaction with PHC facilities, and reference to known routines, patterns and traditions [35] enables patients and communities to generate their own scripts about the nature of service that may be anticipated. The patient's memory reproduces, sustains and amplifies the anticipation that past patterns will recur in future encounters with PHC services. This means patients with specific medical problems, for example TB-suggestive symptoms, may arrive at a course of action that may be neither desirable nor externally rational, primarily because they may apply selective focus to a limited set of cues—usually negative ones—to inform decisions.

The influence of past experience with health care facilities on patient responses to a particular health problem is reported in studies from resource-constrained settings globally [50, 51]. What we wish to highlight here is that this response must be seen as intrinsically relational. It becomes centred on engagement and disengagement of patient with the PHC facility, and simultaneous deliberation with other community members to identify pragmatic possibilities and gain support for decisions that might challenge normative patterns of action [35]. In the process, patients reflect and consider new possibilities and courses of action through imaginative engagement about likely outcomes of such actions. For example, patients were seen to distance themselves from seeking formal health care when they perceived that such action would negatively impact their social identity, for example through possible breach of their privacy and confidentiality, which would expose them to damaging stigma associated with TB or HIV. This pattern has also been reported elsewhere [34, 52], and again is associated with delay in seeking treatment.

In conclusion, PHC systems, which act as a fundamental entry point to formal health care, have salient problems that seem to undermine the quality of health care services provided. This paper has framed treatment seeking, including for symptoms suggestive of TB, within the interplay of the health delivery system challenges and the agency of patients coping with emergent illnesses vis-à-vis the health-system challenges. Negative experiences in PHC facilities sustain “ripple effect” on the subsequent treatment-seeking patterns of patients and community members at large. Individuals who have symptoms of TB can thus be pushed into remaining u-investigated in the community for prolonged periods before fully completing a diagnosis working through formal health care channels. Dealing with TB in TB- and HIV-endemic but resource poor settings requires understanding extant challenges in health care systems, and how these shape treatment seeking behaviour for individual patients. An ‘integrative health care’ model at PHC level is indicated, that employs an interdisciplinary team approach that pursues mutual respect and a vision of health care that permits practitioners and patients to contribute their particular knowledge within a collaborative plan of care [53]. It is worth noting, however, that these findings pertain to a phase of immense economic difficulty in Malawi, which may raise questions about the applicability of the findings in different economic scenarios. Having said this, Malawi is a known low-income country, and besides, for a qualitative study of this nature whereby findings pertain to a specific case context, it is deemed possible to transfer the findings to other settings that are inherently similar.
